# Author Correction: hnRNP H/F drive RNA G-quadruplex-mediated translation linked to genomic instability and therapy resistance in glioblastoma

**DOI:** 10.1038/s41467-021-23484-3

**Published:** 2021-05-12

**Authors:** Pauline Herviou, Morgane Le Bras, Leïla Dumas, Corinne Hieblot, Julia Gilhodes, Gianluca Cioci, Jean-Philippe Hugnot, Alfred Ameadan, François Guillonneau, Erik Dassi, Anne Cammas, Stefania Millevoi

**Affiliations:** 1 Cancer Research Center of Toulouse (CRCT), INSERM UMR 1037, 31037 Toulouse, France; 2 Université Toulouse III Paul Sabatier, 31330 Toulouse, France; 3 Laboratoire d’Excellence “TOUCAN”, Toulouse, France; 4 Institut Universitaire du Cancer de Toulouse-Oncopole, 31100 Toulouse, France; 5 TBI, Université de Toulouse, CNRS, INRA, INSA, Toulouse, France; 6 INSERM U1051, Institute for Neurosciences, Hôpital Saint Eloi, Université de Montpellier 2, 34090 Montpellier, France; 7 Plateforme Protéomique 3P5, Université de Paris, Inserm U1016-institut Cochin, Labex GReX, 22 rue Méchain, 75014 Paris, France; 8 Department of Cellular, Computational and Integrative Biology (CIBIO), University of Trento Via Sommarive 9, 38123 Trento, Italy

**Keywords:** RNA-binding proteins, RNA

Correction to: *Nature Communications* 10.1038/s41467-020-16168-x, published online 27 May 2020.

The original version of this Article contained an error in the “Methods” section, which incorrectly omitted at the start of the “GBM tumour sample” paragraph the following information and reference to previous work: “The used protein extracts derived from both low and high grade gliomas were originally processed and used in the article from from Pr JP Hugnot’s lab^68^”. Additionally, the original version of this Article omitted the reference 68 to the previous work in “Leventoux N, Augustus M, Azar S, et al. Transformation Foci in IDH1-mutated Gliomas Show STAT3 Phosphorylation and Downregulate the Metabolic Enzyme ETNPPL, a Negative Regulator of Glioma Growth. *Sci Rep*. 2020;10(1):5504. Published 2020 Mar 26. 10.1038/s41598-020-62145-1”. This has been added as a reference 68 in the newly corrected paragraph in the “Methods” section under “GBM tumour sample”. These have been corrected in both the PDF and HTML versions of the Article.

