# Supplementary material for: hnRNP H/F drive RNA G-quadruplex-mediated translation linked to genomic instability and therapy resistance in glioblastoma
Source: Nat Commun. 2020 May 27;11:2661. doi: 10.1038/s41467-020-16168-x (PMC7253433; doi:10.1038/s41467-020-16168-x)
Supplement: Supplementary file 1 — Supplementary Information [file 41467_2020_16168_MOESM1_ESM.pdf]

Supplementary Information

hnRNP H/F drive RNA G-quadruplex-mediated translation linked to genomic instability and therapy resistance in glioblastoma

Herviou, Le Bras et al.

Supplementary Figures (1-14) and Supplementary Tables (1-3)

Supplementary Figure 1

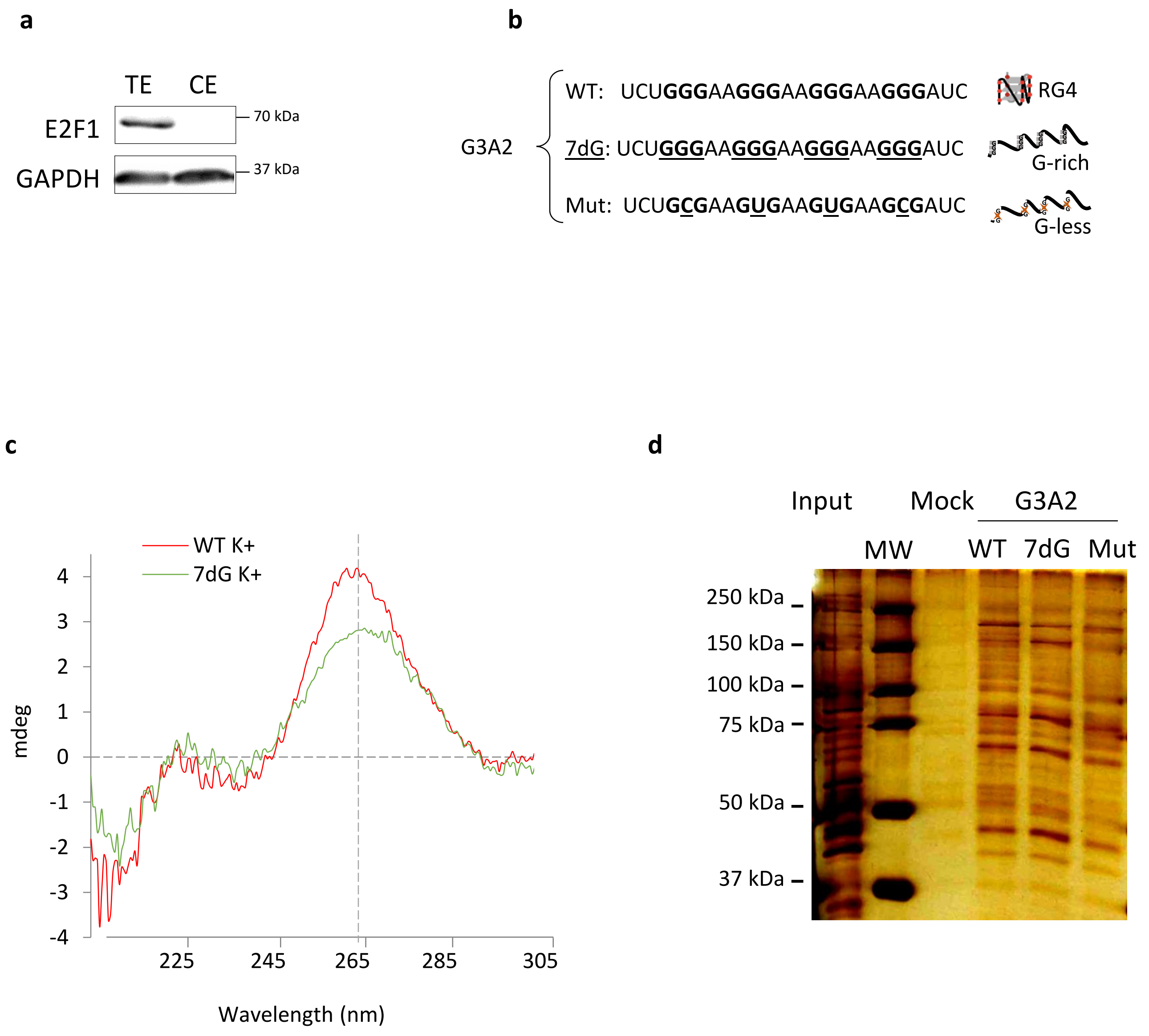

Supplementary Figure 1 (related to Figure 1): G3A2 structuration and protein binding. (a) Subcellular fractionation of U251 GBM cell line followed by western blot analysis of E2F1 (nuclear marker) and GAPDH in the total extract (TE) and cytoplasmic extract (CE). Shown is a representative result from n=3 independent experiments. **(b)** RNAs used in affinity chromatography experiments, containing the G3A2 sequence either native (WT), or 7-deaza-modified (7dG), unable to form RG4s but preserving G-tracts, or mutated (Mut) to both disrupt G-tracts and hamper RG4 formation. **(c)** Circular dichroism spectra of G3A2 WT and 7dG. Each RNA was heat-denatured and allowed to fold at 25°C in 10 mM Tris-Cl (pH 7.5), 0.1 mM EDTA and 100 mM KCl (K+) (WT, red; 7dG, green). **(d)** RNA affinity chromatography using the G3A2, 7dG or Mut RNAs (depicted in **(b)**) incubated with U251 cytoplasmic cell extracts, followed by silver staining. Shown is a representative result from n=4 independent experiments. Source data are provided as a Source Data file.

Supplementary Figure 2

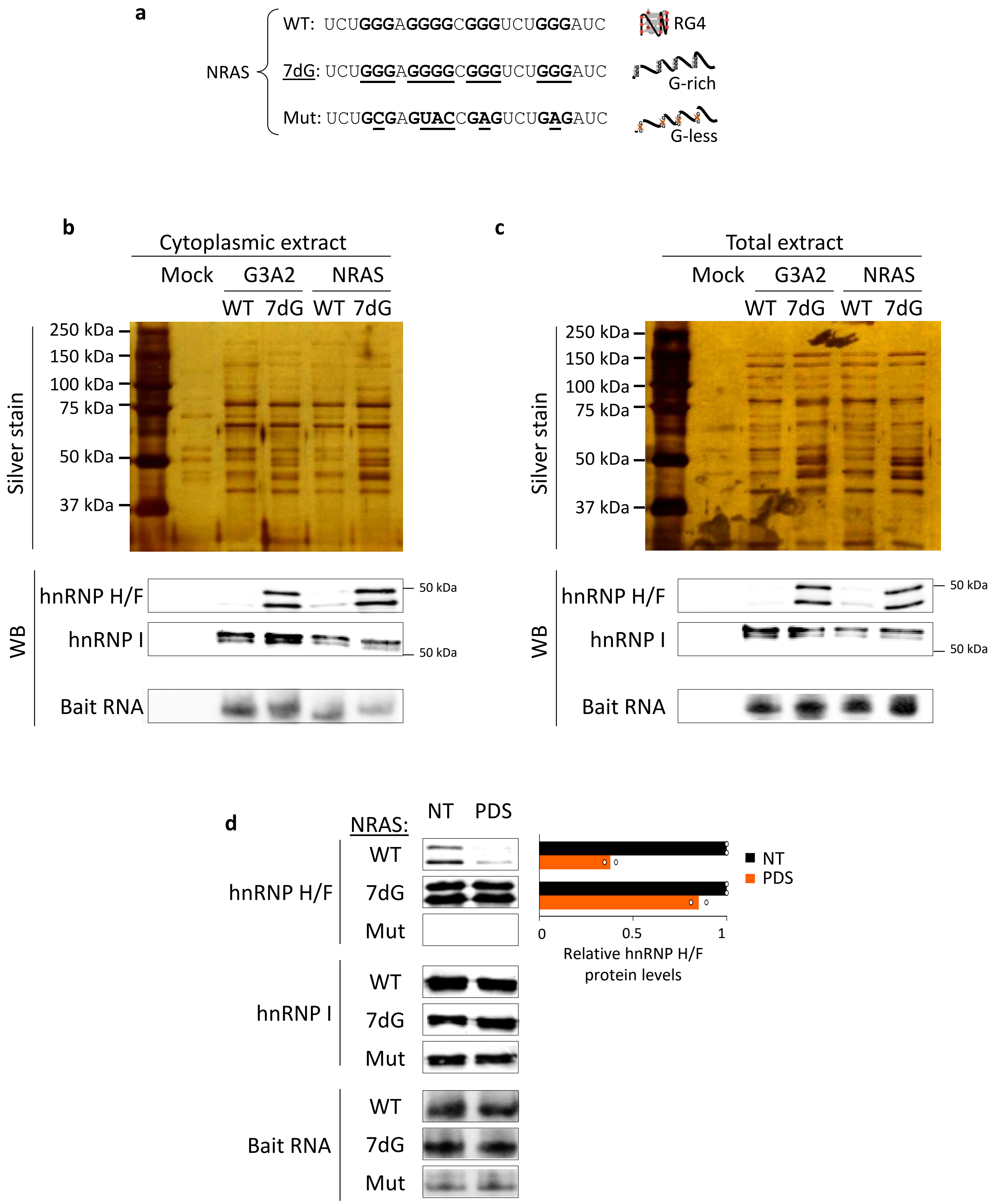

Supplementary Figure 2 (related to Figure 1). G3A2 and NRAS share similar RG4 sequence/structure protein binding, including hnRNP H/F. (a) RNAs used in affinity chromatography experiments, containing the NRAS RG4 sequence either native (WT), or 7-deaza-modified (7dG), or mutated (Mut).

(b,c) RNA affinity chromatography using the G3A2 or NRAS WT or 7dG (depicted in **(a)**) incubated with U251 cytoplasmic **(b)** or total **(c)** cell extracts, followed by silver staining and western blot analysis. Bait RNA: RNAs retained on beads. For **(b, c)** panels, shown is a representative result from n=3 independent experiments. Source data are provided as a Source Data file. **(d)** RNA affinity chromatography using the NRAS RNAs as in **(b,c)**, treated with pyridostatin (PDS) or untreated (NT), followed by western blot analysis, quantification and normalization of the hnRNP H/F protein levels to the control (hnRNP I). Images were acquired with the same exposure time and are representative of n=2 independent experiments. Quantification was obtained from n=2 independent experiments. Source data are provided as a Source Data file.

Supplementary Figure 3

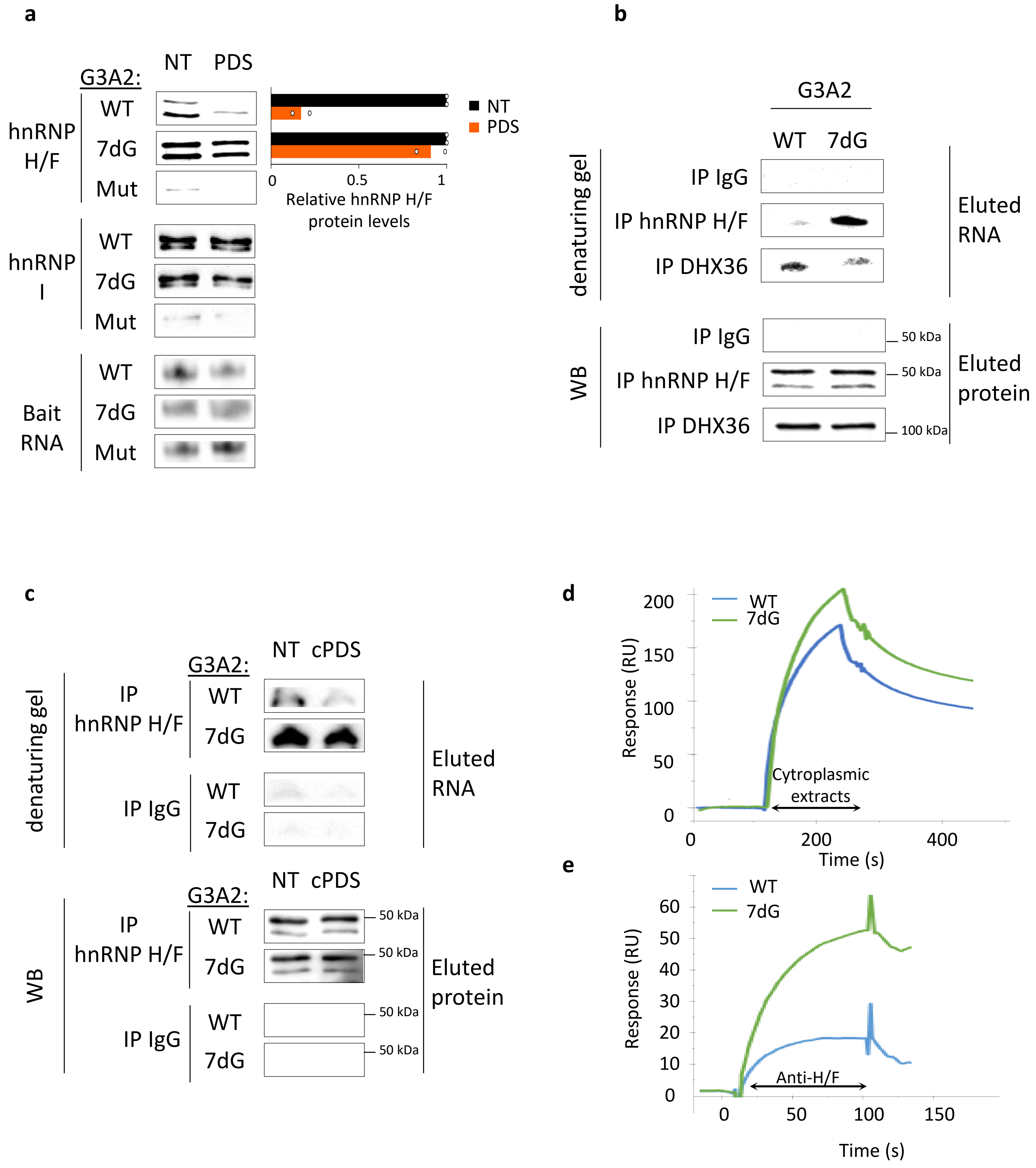

Supplementary Figure 3 (related to Figure 1). RG4 structuration-dependent binding of hnRNP H/F to the G3A2 RNA. **(a)** RNA affinity chromatography using the G3A2 RNAs WT, 7dG or Mut, treated with pyridostatin (PDS) or untreated (NT), followed by western blot analysis, quantification and normalization of the hnRNP H/F protein levels to the control (hnRNP I). Images were acquired with the same exposure time and are representative of n=2 independent experiments. Quantification was obtained from n=2 independent experiments. Source data are provided as a Source Data file.

(b) Immunoprecipitation of hnRNP H/F or DHX36 with the G3A2 WT or 7dG, followed by detection of eluted RNAs using acrylamide denaturing gels and of eluted proteins using western blot analysis. Shown is a representative result from n=4 and n=2 independent experiments for H/F and DHX36 IP respectively. Source data are provided as a Source Data file. **(c)** Immunoprecipitation of hnRNP H/F with the G3A2 WT or 7dG pre-incubated with carboxypyridostatin (cPDS), followed by detection of eluted RNAs using acrylamide denaturing gels or the eluted proteins using western blot analysis. Source data are provided as a Source Data file. **(d-e)** Surface plasmon resonance sensorgrams of U251 cytoplasmic cell lysates on sensor chips coated with the G3A2 WT or 7dG RNA **(d)** and after the addition of hnRNP H/F antibodies **(e)**. mRNA-ribonucleoprotein complexes were formed with both RNAs **(d)** but those formed with the G3A2 7dG preferentially associated with hnRNP H/F **(e)**. Representative of 2 experiments. RU, resonance unit.

Supplementary Figure 4

a

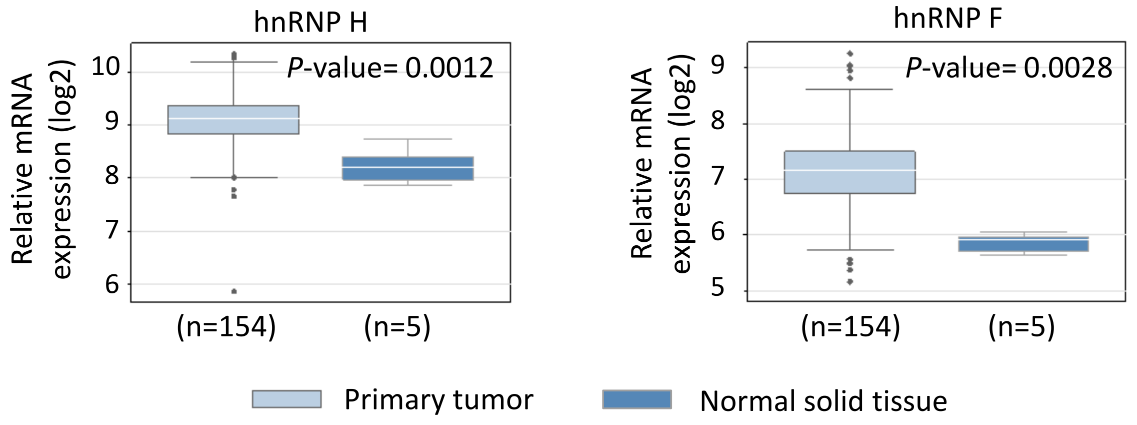

b

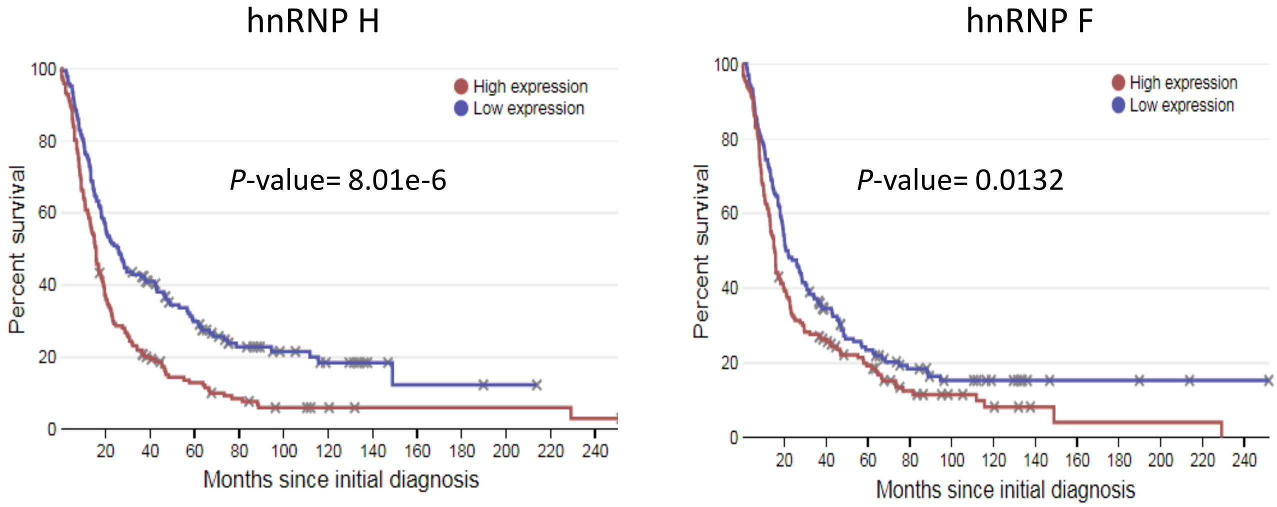

c

CELL LINE	γ-irradiation	TMZ	EGFR	PTEN	p53	MGMT
U251	Sensitive	Sensitive	WT	Mut	Mut	Methylated
LN18	Resistant	Resistant	WT	WT	Mut	WT
U87	Resistant	Sensitive	WT	Mut	WT	Methylated

Supplementary Figure 4 (related to Figure 2). High hnRNP H/F expression in GBM is correlated with poor survival. (a) hnRNP H and F mRNA expression using TCGA data in normal (n=5) and GBM primary tumor (n=154). The band inside the box shows the median and the whiskers show the upper and lower extremes (Two sided non-parametric Mann-Whitney test and Benjamini-Hochberg procedure was used for multiple comparisons). **(b)** Representation of Kaplan-Meier Survival curve using the REMBRANDT database which collects data from 329 brain tumors generated with AffymetrixHGU133v2.0plus using the BETASTASIS resource. **(c)** Chemo-/radio- sensitivity and molecular characteristics of glioblastoma cell lines.

Supplementary Figure 5

a

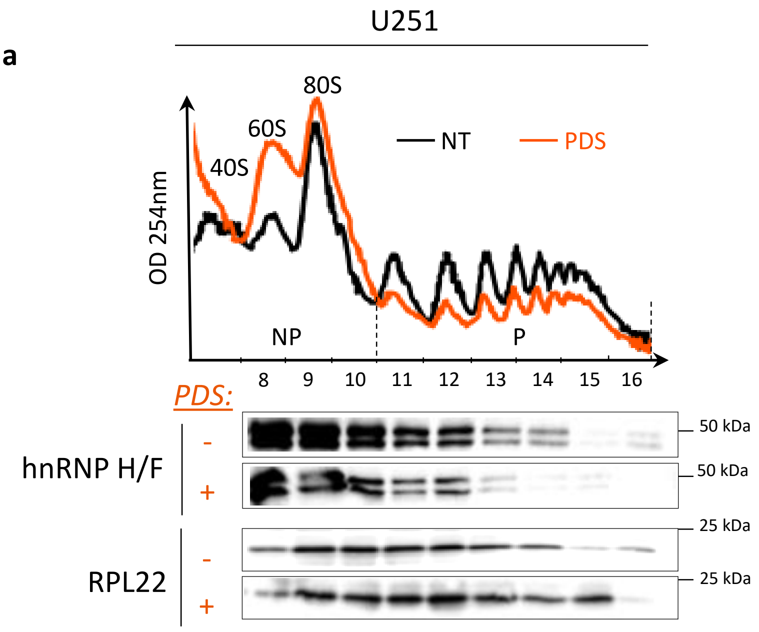

b

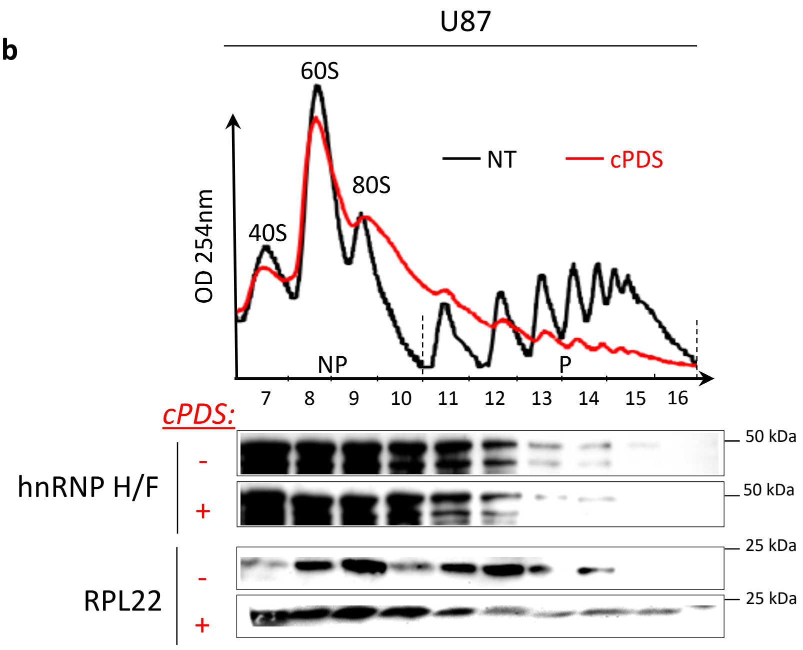

Supplementary Figure 5 (related to Figure 2). RG4 ligand-dependent dissociation of hnRNP H/F from translating ribosomes. (a) Polysome profile of U251 cells untreated (NT) or treated with 20 µM pyridostatin (PDS) for 1 h, followed by Western blot analysis from individual non-polysomal (NP) and polysomal (P) fractions by probing for hnRNP H/F or RPL22 (control). Shown is a representative result from n=3 independent experiments. Source data are provided as a Source Data file. **(b)** As in **(a)**, except with U87 cells treated with 20 µM carboxypyridostatin (cPDS) for 1 h. Source data are provided as a Source Data file. Shown is a representative result from n=3 independent experiments.

Supplementary Figure 6

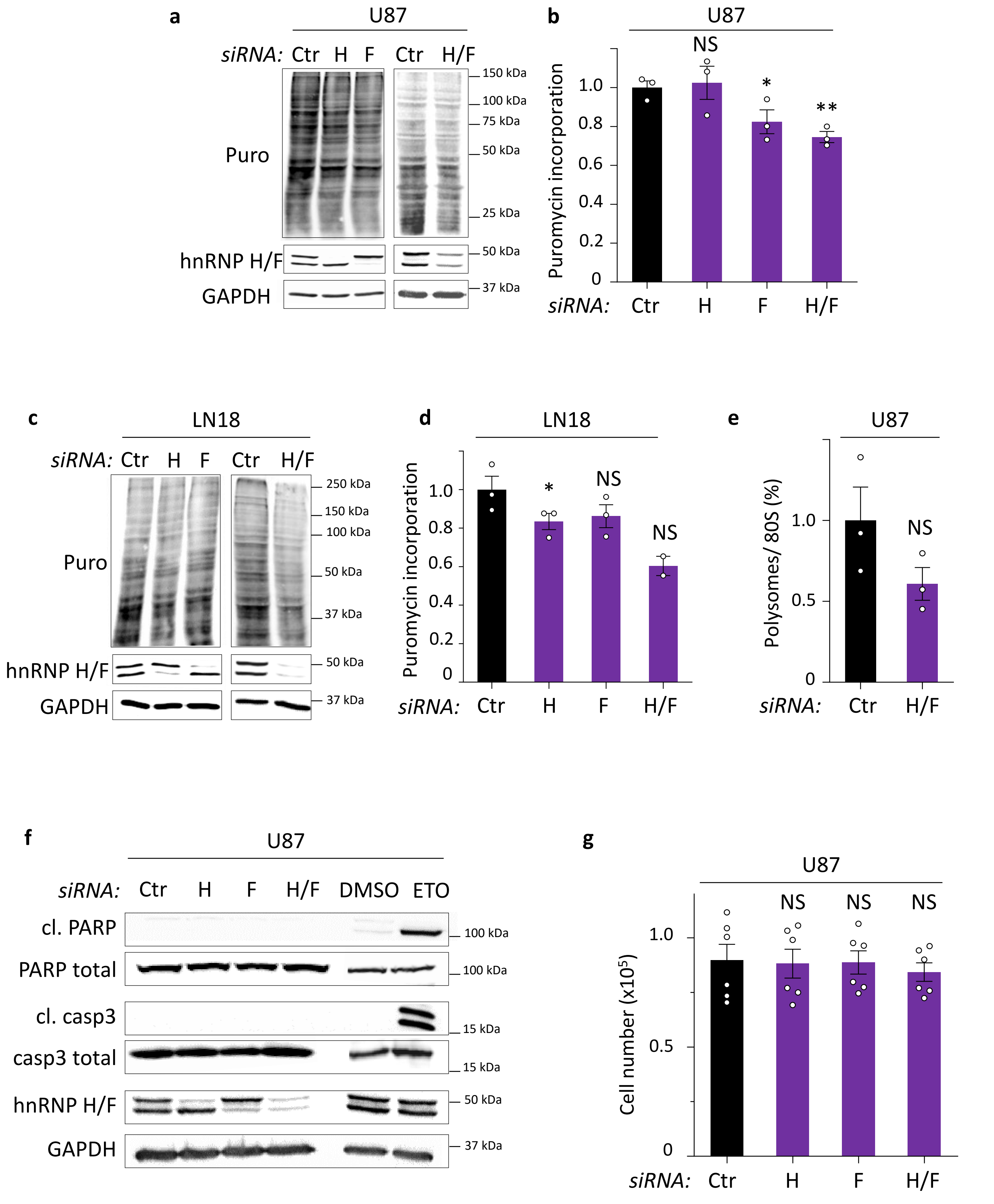

Supplementary Figure 6 (related to Figure 3). hnRNP H/F do not impact on global translation, proliferation or apoptosis. (a) *De novo* protein synthesis analysis by SUnSET assay in U87 cells treated with control (siCtr), hnRNP H and/or hnRNP F (siH, siF or siH/F) siRNAs, followed by western blot analysis of the incorporated puromycin, hnRNP H/F and GAPDH.

(b) Quantification of puromycin incorporation from A. (Data are presented as mean values ± SEM of n=3 independent experiments, *P*-value= 0.0178 and *P*-value= 0.002 for the siF and siH/F respectively, NS, Non Significant (two-sided paired t-test)). **(c,d)** As in **(a)**, except that LN18 cells were used. Data are presented as mean values ± SEM of n=3 for siH and siF and n=2 for siHF independent experiments, *P*-value= 0.046 for the siH, NS, Non Significant (two-sided paired t-test). **(e)** Quantification of the ratio between the area under the HP curve and the 80S area from the polysome profile Figure **3(a)** in the control (siCtr) and hnRNP H/F-depleted (si H/F) conditions. Data are presented as mean values ± SEM of n=3 independent experiments, NS: Non Significant (two-sided paired t-test). **(f)** Western Blot analysis of cleaved caspase 3 (cl. Casp3), cleaved PARP (cl. PARP) and hnRNP H/F expression in U87 cells treated with control (siCtr), hnRNP H and/or hnRNP F (siH, siF or siH/F) siRNAs. Etoposide: positive control. Shown is a representative result from n=3 independent experiments. **(g)** U87 cell number after treatments with control (siCtr), hnRNP H and/or hnRNP F (siH, siF or siH/F) siRNAs. Data are presented as mean values ± SEM of n=6 independent experiments for U87, NS: Non significant (two-sided paired t-test). **(a-g)** Source data are provided as a Source Data file.

Supplementary Figure 7

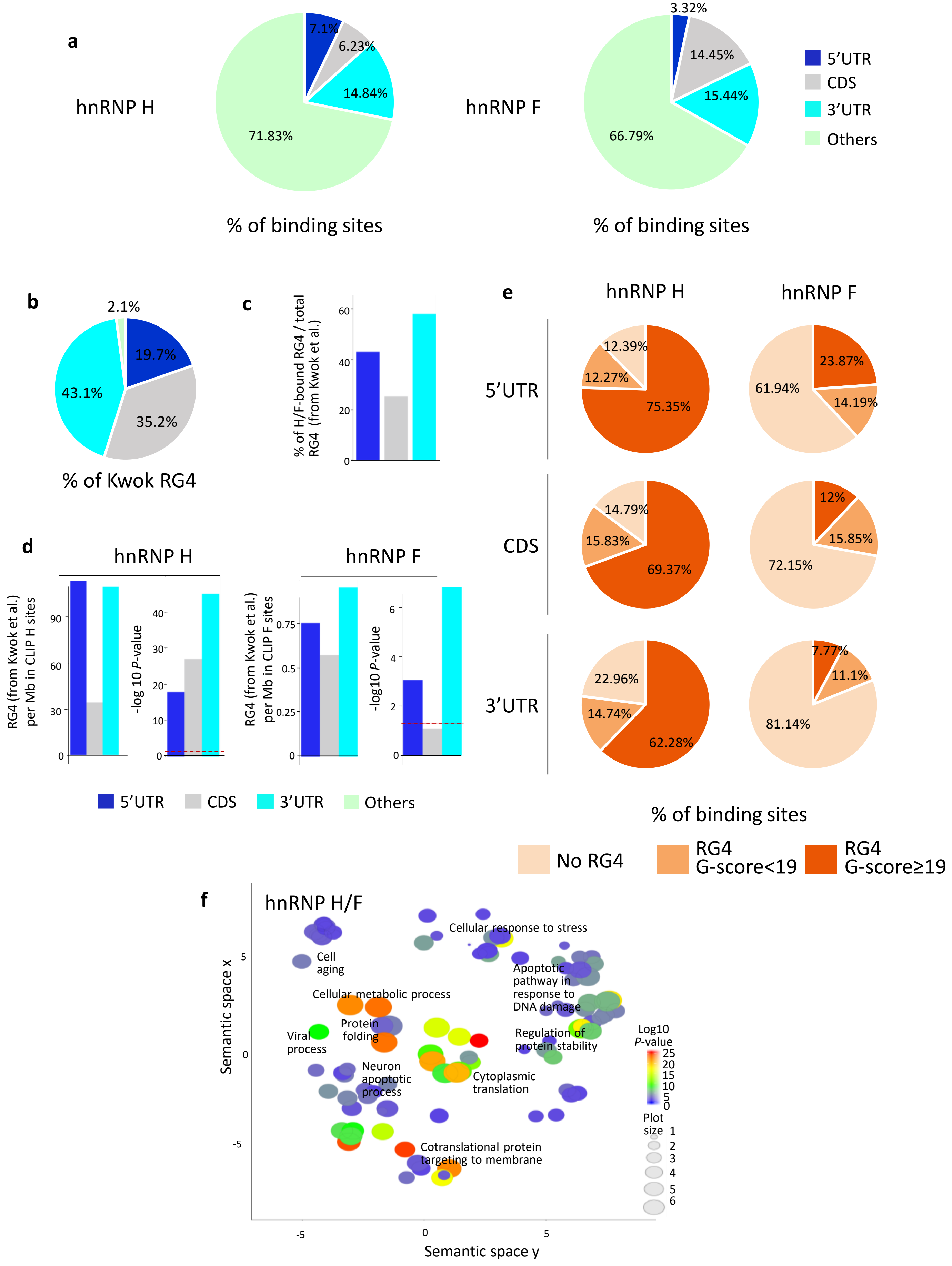

Supplementary Figure 7 (related to Figure 3). hnRNP H/F-RG4 interactions regulate mRNA translation of stress-response genes. (a) Distribution of hnRNP H and F binding sites in genomic region types.

(b) Distribution of experimentally identified RG4s from [1]. **(c)** Percentage of RG4s in hnRNP H/F CLIP sites over all RG4s experimentally validated (based on [1]) for 5'UTR, CDS, and 3'UTR regions. **(d)** Density of RG4s per Mb of hnRNP H and hnRNP F binding sites, along with the -log10 *P*-value of the enrichment with respect to random sites (iii). **(e)** Proportion of hnRNP H and F binding sites in 5'UTR, CDS, and 3'UTR containing RG4/RG4-high elements. **(f)** Gene ontology enrichment analysis of the common targets of hnRNP H/F extracted from CLIP analysis [2,3] and plotted with REVIGO. Bubble plot sizes indicate the generality of GO terms. Larger bubbles relate to general terms whereas smaller bubbles imply more specific GO terms.

Supplementary Figure 8

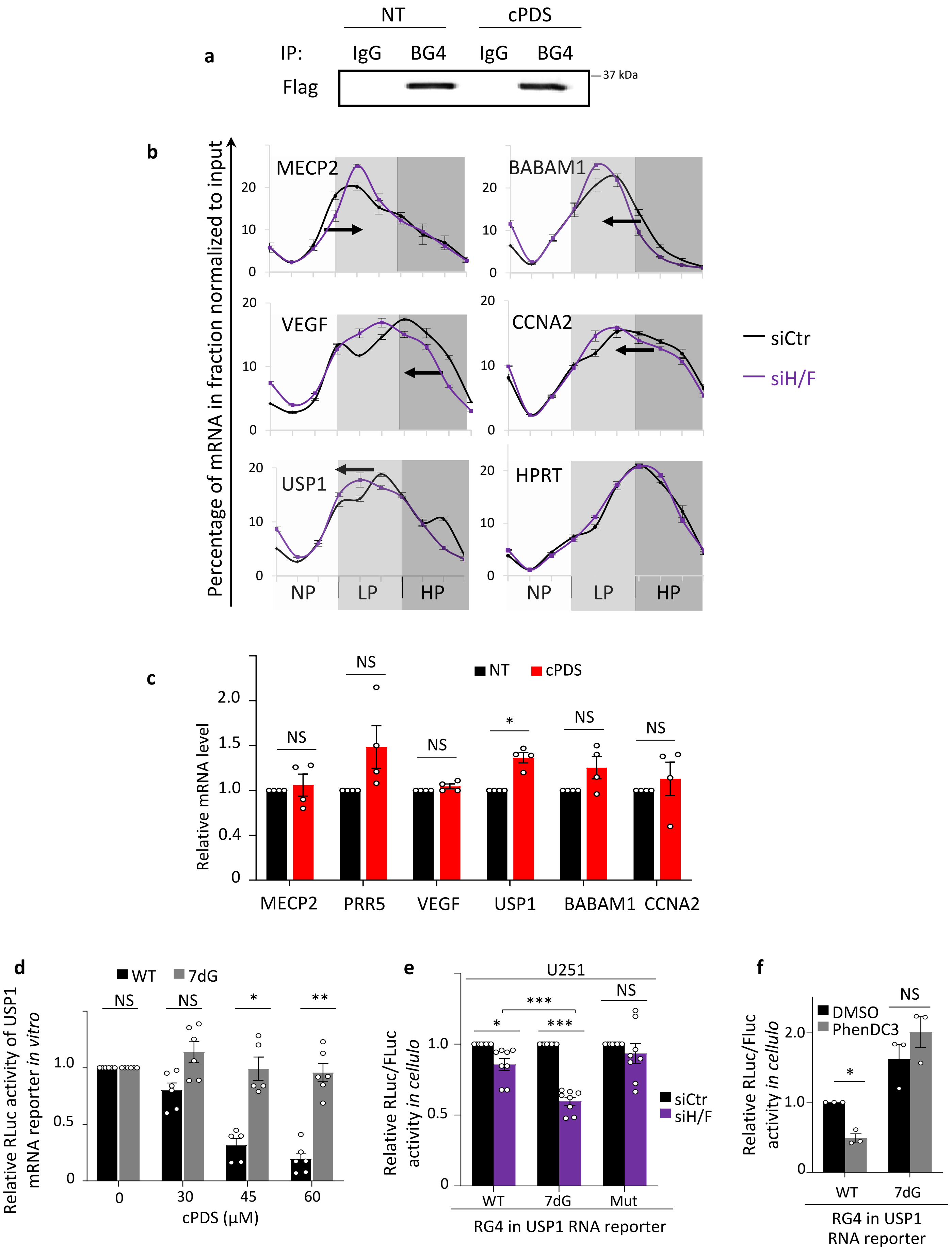

Supplementary Figure 8 (related to Figure 3). hnRNP H/F drive mRNA translation of stress-response genes.

(a) Immunoprecipitation of *in cellulo* RNA-protein complexes (RIP) in U87 cells (cytoplasmic fraction), untreated (NT) or treated with 20 µM cPDS for 2 h, using the Flag-tagged BG4 antibody or control IgG, followed by western blot analysis of the Flag epitope tag. Shown is a representative result from n=3 independent experiments. **(b)** RT–qPCR analysis from individual NP, LP, HP fractions extracted from the polysome profile in **Figure 3(a)** using specific primers for the indicated mRNAs. Data are presented as mean values ± SEM of n=3 independent experiments, Source data are provided as a Source Data file. **(c)** Quantitative RT-qPCR using specific primers for the indicated mRNAs and input mRNAs extracted from U87 cells untreated (NT) or treated with 20 mM carboxypyridostatin (cPDS) for 1 h. Data were plotted relatively to HPRT mRNA amounts. Data are presented as mean values ± SEM of n=4 independent experiments, **P*-value= 0.008 for USP1, NS: Non Significant (two-sided paired t-test). Source data are provided as a Source Data file. **(d)** *In vitro* translation in RRL of *in vitro* transcribed USP1 reporter mRNAs (capped and polyadenylated) containing the RG4 unmodified (WT), 7dG-modified (7dG) with increasing amounts of carboxypyridostatin (cPDS), as indicated. **(e,f)** Ratio of Renilla/Firefly luciferase activities (Rluc/Fluc) determined using U251 cells treated with control (siCtr) and hnRNP H/F (siH/F) siRNAs **(e)** or 10 µM PhenDC3 for 16 h **(f)**, followed by cotransfection with USP1 RNA reporters containing the RG4 unmodified (WT), 7dG-modified (7dG) or mutated (Mut) and an internal control mRNA encoding the Firefly luciferase (Fluc). **(d-f)** Data are presented as mean values ± SEM of n=3 to n=6 independent experiments *P<0.05, **P<0.005, ***P<0.0005, NS: Non Significant (two-sided paired t-test and one-sided paired t-test for **(d,e)** and **(f)** respectively). Source data and exact *P*-values are provided as a Source Data file.

.

Supplementary Figure 9

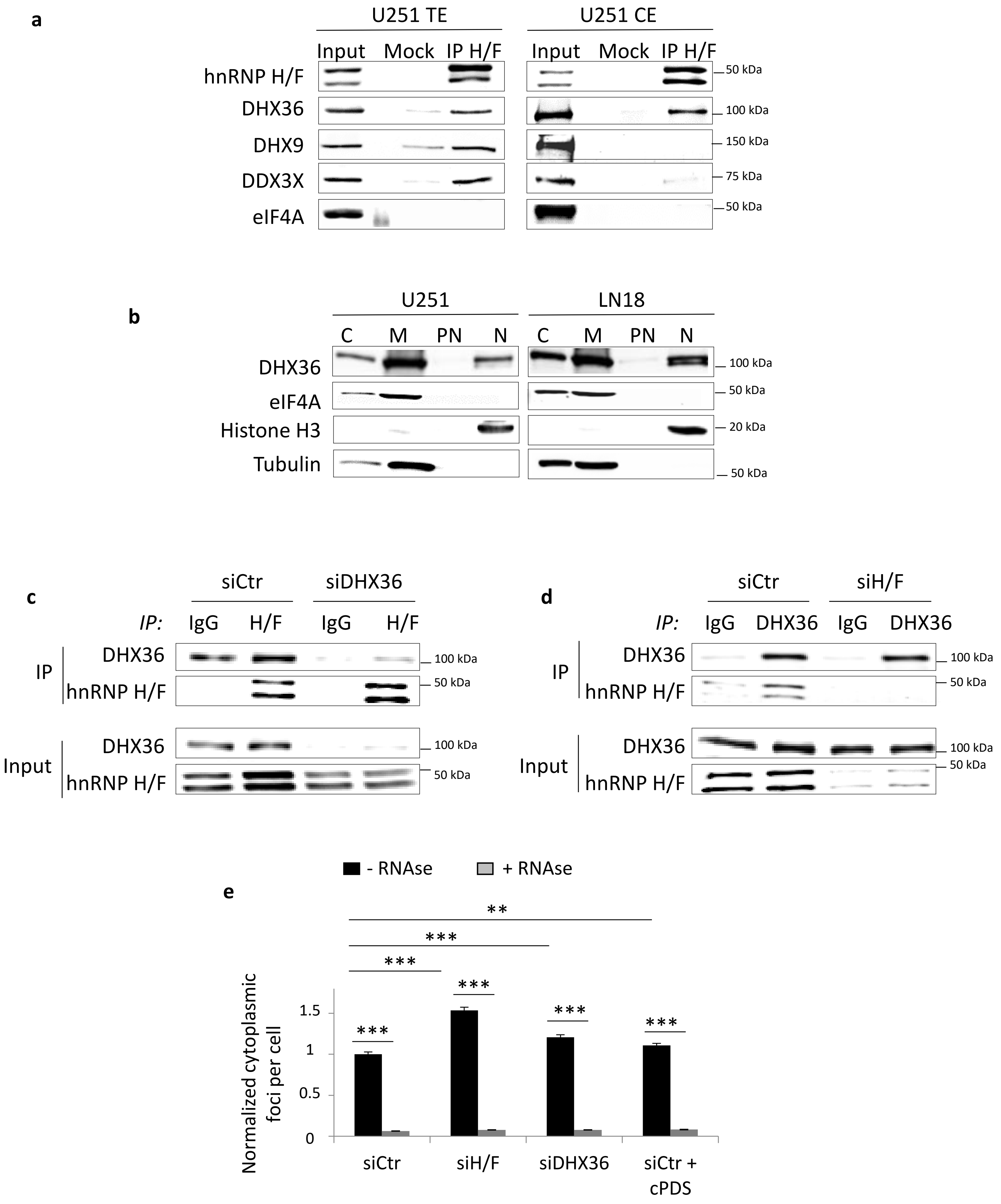

Supplementary Figure 9 (related to Figure 4). hnRNP H/F and DHX36 directly interact in the cytoplasm compartment. (a) Immunoprecipitation (IP) of U251 total (TE) or cytoplasmic (CE) extracts, followed by western blot analysis and probing with the indicated antibodies. Shown is a representative result from n=3 independent experiments. Source data are provided as a Source Data file.

(b) Subcellular fractionation of U251 and LN18 cell lines followed by western blot analysis of DHX36, eIF4A and tubulin (cytoplasmic and microsomal marker), histone H3 (nuclear marker). Nuclear (N), microsomal (M), perinuclear (PN) and cytosolic fractions (C). Shown is a representative result from n=3 independent experiments. **(c,d)** IP of *in cellulo* RNA-protein complexes in cytoplasmic extracts from U87 cells with the hnRNP H/F or DHX36 antibody, after treatment with control (siCtr) siRNAs and either DHX36 (siDHX36) **(c)** or hnRNP H/F (siH/F) **(d)** siRNAs, followed by western blot analysis. Shown is a representative result from n=4 independent experiments. **(e)** Immunofluorescence experiments in U251 cells using the BG4 antibody after treatment with control (siCtr), hnRNP H/F (siH/F), DHX36 (siDHX36) siRNAs and cPDS. Quantification of cytoplasmic BG4 foci number per cell and data are presented as mean values ± SEM. Number of cells counted in the -RNase conditions: 1321 cells for siCtr, 1811 cells for siH/F, 1953 cells for siDHX36, 2103 cells for siCtr+cPDS ; Number of cells counted in the +RNase conditions: 1985 cells for siCtr, 1981 cells for siH/F, 1954 cells for siDHX36, 2347 cells for siCtr+cPDS. Statistical significance was performed on the full cell populations $^*P<0.05$, $^{**}P<0.005$, $^{***}P<0.0005$, NS: Non-Significant (two-sided Kolmogorov–Smirnov test). Source data and exact *P*-values are provided as a Source Data file.

Supplementary Figure 10

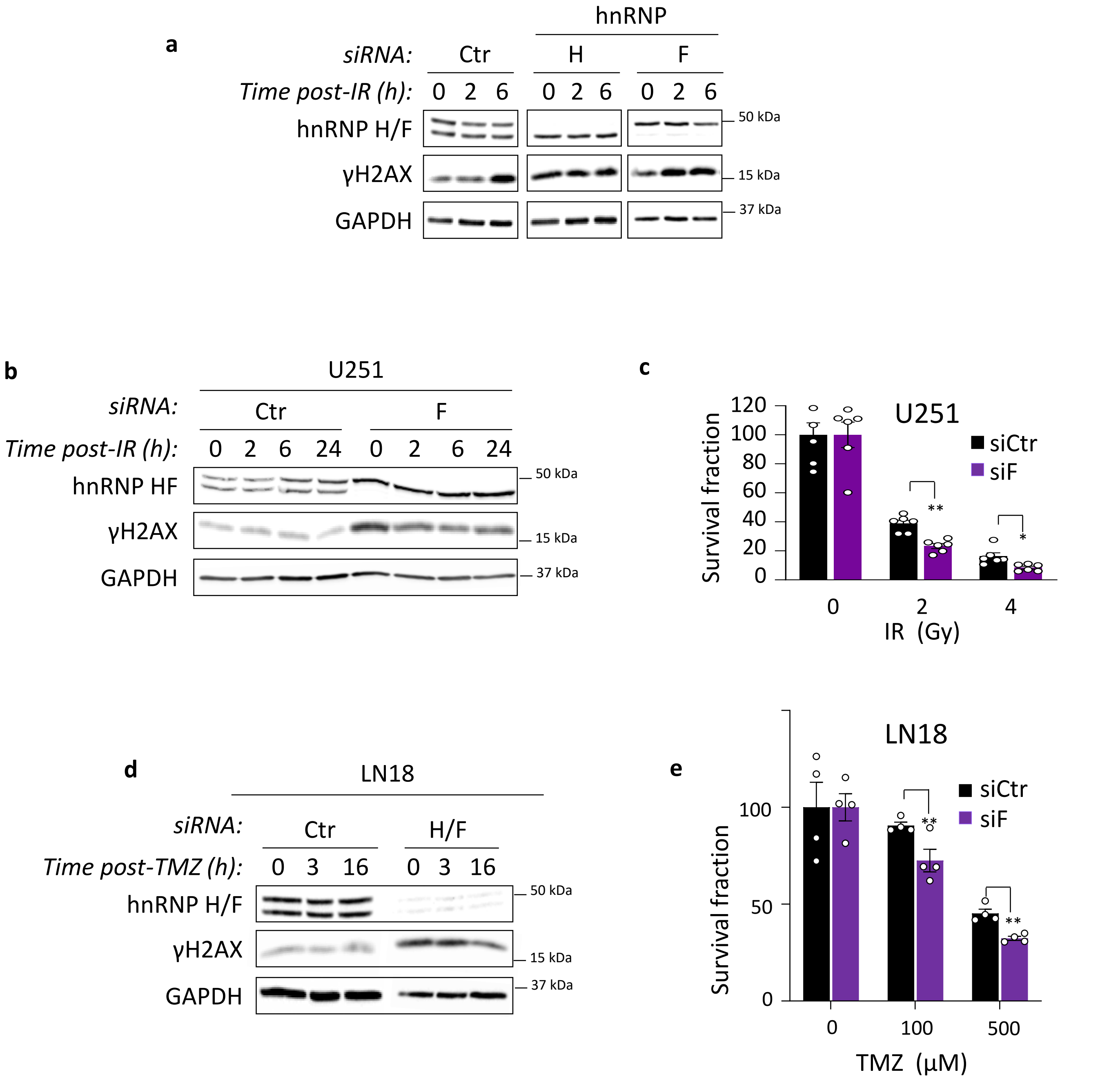

Supplementary Figure 10 (related to Figure 5). hnRNP H/F silencing increases GBM cell radio- and chemo-sensitivity. (a) Quantification of DNA repair kinetics by western blot analysis of γ-H2AX after 4 Gy γ-irradiation in LN18 cells treated with control (siCtr), hnRNP H or hnRNP F (siH or siF) siRNAs. Shown is a representative result from n=2 independent experiments. Source data are provided as a Source Data file. **(b)** Quantification of DNA repair kinetics by western blot analysis of γ-H2AX after 4Gy γ-irradiation in U251 cells treated with control (siCtr) or hnRNP F (siF) siRNAs. Shown is a representative result from n=3 independent experiments. **(c)** Plating efficiency assays measuring the cell survival fraction in U251 treated with control (siCtr) or hnRNP F (siF) siRNAs and submitted to a radiation dose scale. Data are presented as mean values ± SEM of 6 wells, *P*-value= 0.0026 and *P*-value= 0.0378 for the 2 Gy and 4 Gy dose respectively (two-sided paired t-test). **(d)** As in **(a)**, except that cells were treated with 500 µM temozolomide (TMZ). Shown is a representative result from n=3 independent experiments. Source data are provided as a Source Data file.

(e) Plating efficiency assay measuring the cell survival fraction in LN18 treated with control (siCtr) or hnRNP F (siF) siRNAs and submitted to temozolomide (TMZ) dose scale. Data are presented as mean values ± SEM of 4 wells, *P*-value= 0.0005 and *P*-value= 0.0014 for the 100 µM and 500 µM dose respectively (two-sided paired t-test). Source data are provided as a Source Data file.

Supplementary Figure 11

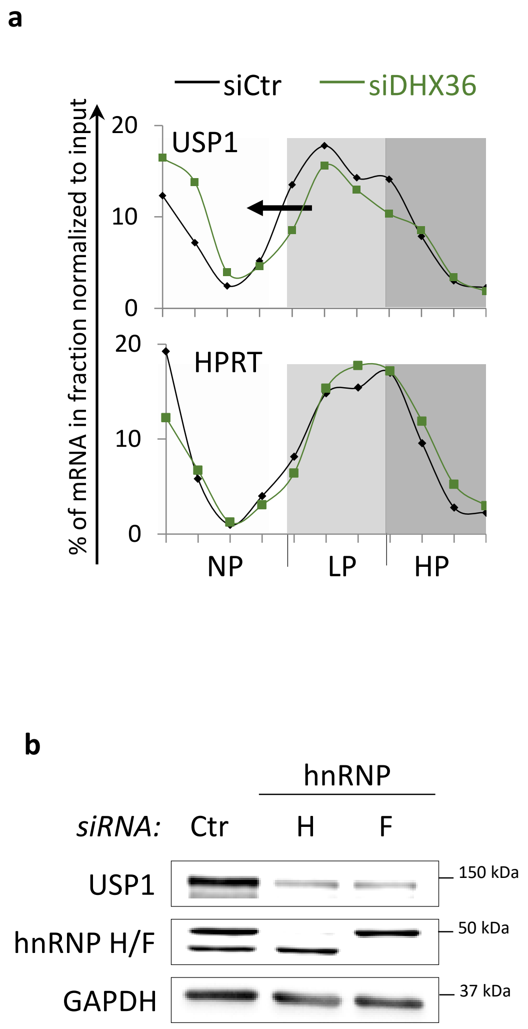

Supplementary Figure 11 (related to Figure 6). DHX36 and both hnRNP H and hnRNP F regulate USP1 expression. (a) Polysome profile of U87 cells treated with control (siCtr) and DHX36 (siDHX36) siRNAs, followed by RT–qPCR analysis from individual Non polysomes (NP), Light Polysomes (LP), heavy Polysomes (HP) fractions, using specific primers for DHX36 and HPRT mRNAs, and quantification by measuring the distribution of each mRNA across the gradient. Shown is a representative result from n=2 independent experiments. Source data are provided as a Source Data file. **(b)** Western blot analysis of USP1 in U87 cells treated with control (siCtr), hnRNP H (siH) and hnRNP F (siF) siRNAs. Shown is a representative result from n=2 independent experiments. Source data are provided as a Source Data file.

Supplementary Figure 12

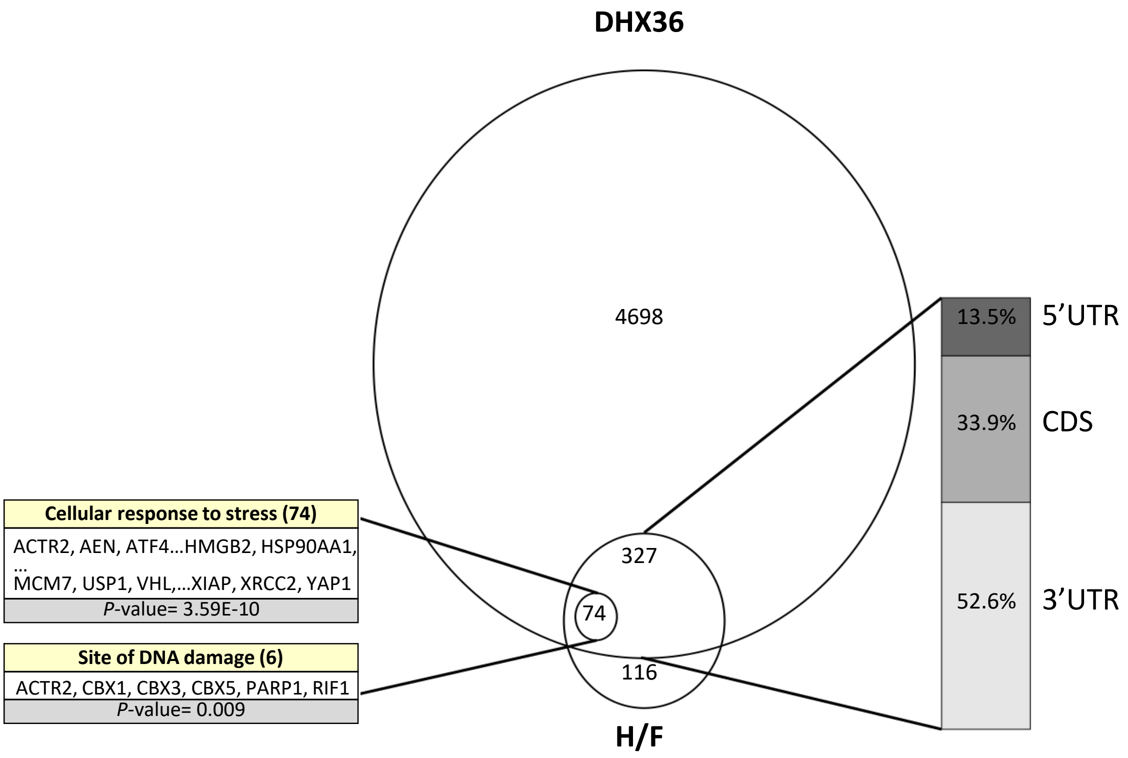

Supplementary Figure 12. Overlap between genes targeted by DHX36 and HNRNP H/F according to CLIP assays. Venn diagram showing unique or common CLIP-derived binding sites between hnRNP H/F and DHX36. Percentages of common genes bound in the different regions composing the mRNA (5'UTR, CDS, and 3'UTR) are shown on the right. Enriched terms related to cellular stress and DNA damage are instead shown on the left, with representative genes annotated to the terms and enrichment *P*-value indicated (Two-sided fisher exact test with Benjamini-Hochberg multiple testing adjustment).

Supplementary Figure 13

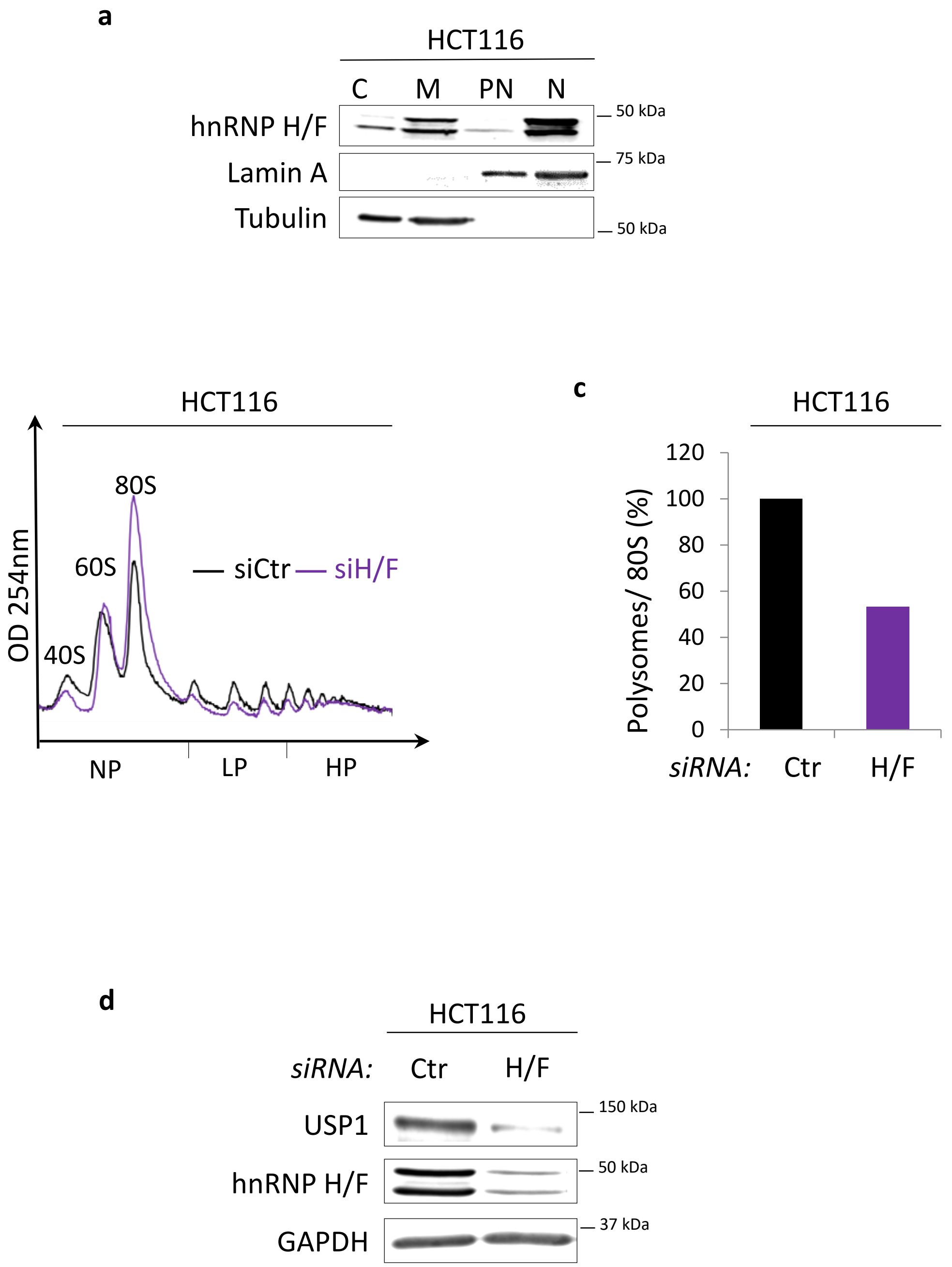

Supplementary Figure 13. hnRNP H/F is a translational regulator in colon cancer cell lines. (a) Subcellular fractionation of HCT116 cell line followed by western blot analysis of hnRNP H/F, lamin A (nuclear marker) and tubulin (cytosolic marker associated to microsomes). Nuclear (N), microsomal (M), perinuclear (PN) and cytosolic fractions (C). Shown is a representative result from n=3 independent experiments. Source data are provided as a Source Data file. **(b)** Polysome profile of HCT116 cells treated with control (siCtr) and hnRNP H/F (siH/F) siRNAs. **(c)** Quantification of the ratio between the area under the HP curve and the 80S area from the polysome profile figure S13 **(b)** in the control (siCtr) and hnRNP H/F-depleted (si H/F) conditions. **(d)** Western blot analysis of USP1 in HCT116 cells treated with siRNA control (siCtr), siRNAs against hnRNP H/F (siH/F). Shown is a representative result from n=3 independent experiments. Source data are provided as a Source Data file.

Supplementary Figure 14

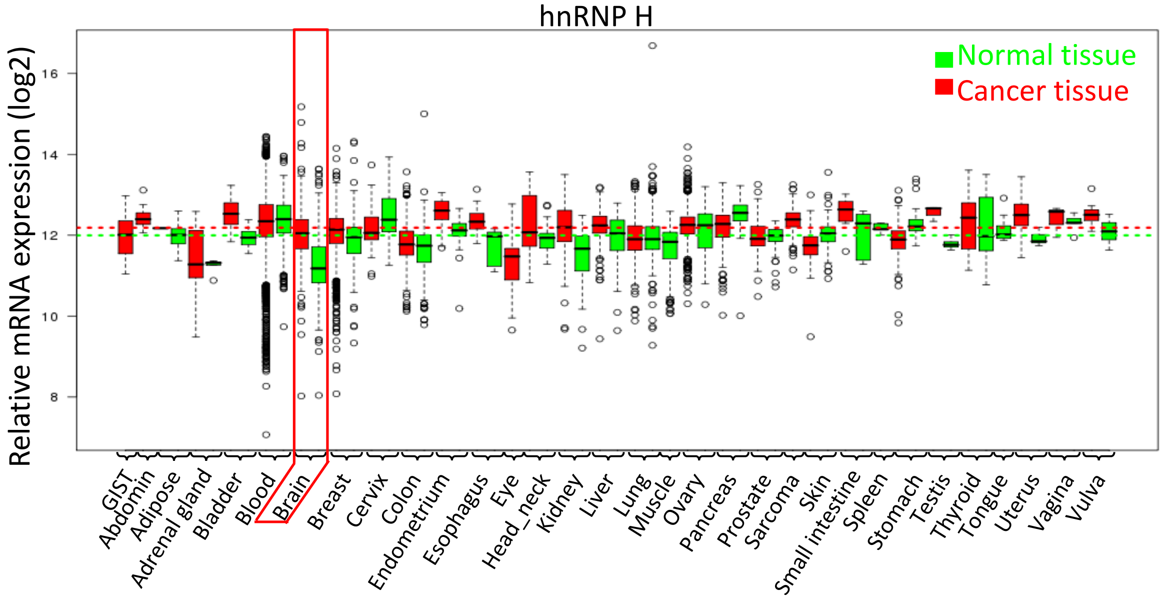

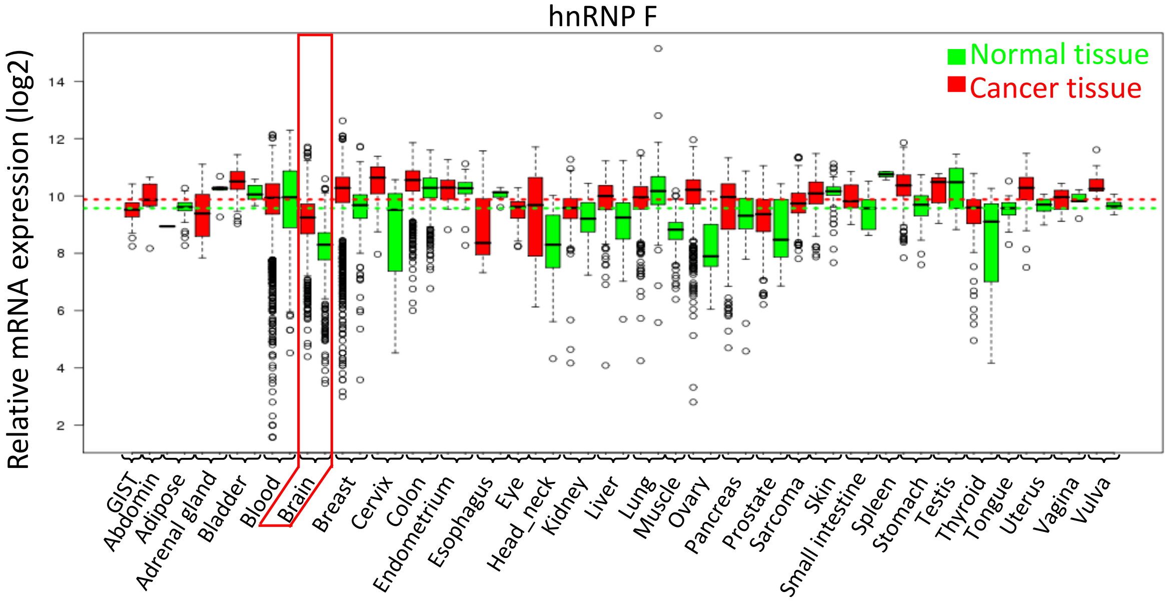

Supplementary Figure 14. hnRNP H/F are deregulated in many tumors. Expression profile of hnRNP H and hnRNP F across diverse human cancers and normal tissues recovered from GENT database using data generated by Affymetrix U133plus2 platforms. The sample size of GENT is >24300 for U133plus2 platforms. The band inside the box shows the mean of the log2 fold change and the whiskers show the upper and lower extremes. Statistical test based on expression profile at each tissue. For each tissue, log2 fold change is calculated.

Supplementary Table 1. List of primers for generating the DNA templates used for the synthesis of luciferase reporter mRNAs

Primer name	Primer sequence
G3A2 WT	tgatacTAATACGACTCACTATAGGtcaacttctactctGGGAAGGGAAGGGAAGGGatcatcatgcatcatctcgctagctaa
G3A2 Mut	tgatacTAATACGACTCACTATAGGtcaacttctactctGCGAAGTGAAGTGAAGCGatcatcatgcatcatctcgctagctaa
NRAS WT	tgatacTAATACGACTCACTATAGGtcaacttctactctGGGAGGGGCGGGTCTGGGatcatcatgcatcatctcgctagctaa
NRAS Mut	TgatacTAATACGACTCACTATAGGtcaacttctactctGCGAGTACCGAGTCTGAGatcatcatgcatcatctcgctagctaat

Note: Capital letter: T7 promoter; Capital and underlined letter: RG4 sequences; Underlined: NheI restriction site for transcription run-off.

Supplementary Table 2. List of primers used in RT-qPCR

Primer	Sequence
MECP2_Fw	CGCTCTGCTGGGAAGTATGA
MECP2_Rv	GCTTTGGGAGATTTGGGCTT
PRR5_Fw	CACTTCCGGAATGCCATCAC
PRR5_Rv	CTCCAGGCGCAGGTAGTC
VEGF_Fw	ACTGCCATCCAATCGAGACC
VEGF_Rv	CGGCTTGTCACATTTTTCTTGTC
USP1_Fw	ACAGTCCTTAATCATTTCGGTTGA
USP1_Rv	GGAGTTGGCATGTTTCTTGAATGT
BABAM1_Fw	TGTTCCAGTGCCCATATTTCT
BABAM1_Rv	AACAGTTTCGCCATGCAGTT
HPRT_Fw	TGCTTTCCTTGGTCAGGCAGT
HPRT_Rv	CTTCGTGGGGTCCTTTTCACC
CCNA2_Fw	TGCTGACCCATACCTCAAGT
CCNA2_Rv	GGTAGGTCTGGTGAAGGTCC

Supplementary Table 3. Clinicopathological parameters of the patients and tumors

Designation	Tumor type	1p/19q codeletion	IDH1 mutation	ATRX
**LGG103**	Oligodendroglioma II	Deleted	R132H	preserved
**LGG112**	Oligodendroglioma II	Deleted	R132H	preserved
**LGG110**	Astrocytoma II	no deletion	R132H	loss
**LGG141**	Astrocytoma II	no deletion	R312H	loss
**Gli22b**	GBM			
**Gli25**	GBM			
**Gli26**	GBM			

Online References

1. Kwok CK, Marsico G, Sahakyan AB, Chambers VS, Balasubramanian S. rG4-seq reveals widespread formation of G-quadruplex structures in the human transcriptome. *Nat. Methods* **13**, 841-848 (2016).

2. Braun S, *et al.* Decoding a cancer-relevant splicing decision in the RON proto-oncogene using high-throughput mutagenesis. *Nat Commun* **9**, 3315 (2018).

3. Huelga SC, *et al.* Integrative genome-wide analysis reveals cooperative regulation of alternative splicing by hnRNP proteins. *Cell Rep* **1**, 167-178 (2012).
