# Supplementary material for: hnRNP H/F drive RNA G-quadruplex-mediated translation linked to genomic instability and therapy resistance in glioblastoma
Source: Nat Commun. 2020 May 27;11:2661. doi: 10.1038/s41467-020-16168-x (PMC7253433; doi:10.1038/s41467-020-16168-x)
Supplement: Supplementary file 3 — Description of Additional Supplementary Files [file 41467_2020_16168_MOESM3_ESM.pdf]

Description of Additional Supplementary Files

File Name: Supplementary Data 1

Description: RP-MS analysis of proteins bound to RG4 WT or 7dG

Description of the different sheets (sheet 1). List of proteins bound to RG4 WT or 7dG, FDR<5% and FC>1,5 (sheet 2). List of background proteins bound to RG4 WT and 7dG, FDR>5% and/or FC<1,5 (sheet 3). Heatmap and PCA (sheet 4).

File Name: Supplementary Data 2

Description: Intersection of our RP-MS and the RNA-binding total proteome

Intersection between the proteins bound to RG4 WT or 7dG (from our RP-MS analysis) and RNA-binding proteins identified using RNA interactome capture methods (sheet 1). RBP catalogs extracted from Perrez-Perri et al., Gerstberger et al., Queiroz et al., Trendel et al., Urdaneta et al. (sheet 2).

File Name: Supplementary Data 3

Description: Enrichment of RG4s in the CLIP-derived binding regions of hnRNP H/F relative to random control sequences (sheet 1).
