# Supplementary material for: hnRNP H/F drive RNA G-quadruplex-mediated translation linked to genomic instability and therapy resistance in glioblastoma
Source: Nat Commun. 2020 May 27;11:2661. doi: 10.1038/s41467-020-16168-x (PMC7253433; doi:10.1038/s41467-020-16168-x)
Supplement: Supplementary file 7 — Reporting Summary [file 41467_2020_16168_MOESM7_ESM.pdf]

nature research

Corresponding author(s): Millevoi Stefania

Last updated by author(s): Mar 27, 2020

Reporting Summary

Nature Research wishes to improve the reproducibility of the work that we publish. This form provides structure for consistency and transparency in reporting. For further information on Nature Research policies, see Authors & Referees and the Editorial Policy Checklist.

Statistics

For all statistical analyses, confirm that the following items are present in the figure legend, table legend, main text, or Methods section.

n/a	Confirmed	
☐	☒	The exact sample size (n) for each experimental group/condition, given as a discrete number and unit of measurement
☐	☒	A statement on whether measurements were taken from distinct samples or whether the same sample was measured repeatedly
☐	☒	The statistical test(s) used AND whether they are one- or two-sided<br>*Only common tests should be described solely by name; describe more complex techniques in the Methods section.*
☒	☐	A description of all covariates tested
☐	☒	A description of any assumptions or corrections, such as tests of normality and adjustment for multiple comparisons
☒	☐	A full description of the statistical parameters including central tendency (e.g. means) or other basic estimates (e.g. regression coefficient) AND variation (e.g. standard deviation) or associated estimates of uncertainty (e.g. confidence intervals)
☐	☒	For null hypothesis testing, the test statistic (e.g. F, t, r) with confidence intervals, effect sizes, degrees of freedom and P value noted<br>*Give P values as exact values whenever suitable.*
☒	☐	For Bayesian analysis, information on the choice of priors and Markov chain Monte Carlo settings
☒	☐	For hierarchical and complex designs, identification of the appropriate level for tests and full reporting of outcomes
☒	☐	Estimates of effect sizes (e.g. Cohen's d, Pearson's r), indicating how they were calculated

Our web collection on statistics for biologists contains articles on many of the points above.

Software and code

Policy information about availability of computer code

Data collection

Jasco J-815 spectropolarimeter
StepOne v2.2.2 (Applied Biosystems)
BIAcore T200 optical biosensor instrument (GE Healthcare)
ISCO density gradient fractionation system (Foxy Jr fraction collector coupled to UA-6UV detector, Lincoln, NE) and TracerDAQ data acquisition software 2.3
Clariostar BMG Labtech
Clariostar software v.5.21 R4
Chemikapt N° 06-13460
Harmony(R) v4.8
Operetta CLS – High Content Imaging System PERKIN ELMER

Data analysis	Columbus version 2.8.2<br>Perseus software version 1.6.1.1<br>Inkscape v 0.92.4<br>Gimp v 2.10.18<br>ImageJ v 1.52<br>Microsoft Excel<br>Microsoft power point<br>Graphpad Prism8<br>Revigo (http://revigo.irb.hr/)<br>StepOne software v2.2.2 (Applied Biosystems)<br>BIAevaluation 3.0 software (Biacore AB)<br>MARS Clariostar Analysis Software v.3.20 R2<br>R version 3.6.1<br>R studio version 1.0.153

For manuscripts utilizing custom algorithms or software that are central to the research but not yet described in published literature, software must be made available to editors/reviewers. We strongly encourage code deposition in a community repository (e.g. GitHub). See the Nature Research guidelines for submitting code & software for further information.

Data

Policy information about availability of data

All manuscripts must include a data availability statement. This statement should provide the following information, where applicable:

- Accession codes, unique identifiers, or web links for publicly available datasets
- A list of figures that have associated raw data
- A description of any restrictions on data availability

The mass spectrometry proteomics data have been deposited to the ProteomeXchange Consortium via the PRIDE [1] partner repository with the dataset identifier PXD015609. CLIP data for DHX36, hnRNP H and hnRNP F were obtained from GEO ID GSE105171, E-MTAB-6221, GSE34993, respectively.
The REMBRANDT dataset was available at the Betastasis website (http://www.betastasis.com/glioma/rembrandt/kaplan-meier_survival_curve/)
The source data underlying Fig. 1(d-f), 2(a,b,c) ; 3(a,d,e,f,g) ; 4(a,b,c,d,e) ; 5(a,b,d,e,f) ; 6(b,c,e,f) are provided as a Source Data file
The source data underlying Supplementary Figures 1(d), 2(b-d), 3(a,c), 5(a,b), 6(a-g), 8(b-f), 9(a,e), 10(a,d,e), 11(a,b), 13(a,d).

Field-specific reporting

Please select the one below that is the best fit for your research. If you are not sure, read the appropriate sections before making your selection.

☒ Life sciences ☐ Behavioural & social sciences ☐ Ecological, evolutionary & environmental sciences

For a reference copy of the document with all sections, see nature.com/documents/nr-reporting-summary-flat.pdf

Life sciences study design

All studies must disclose on these points even when the disclosure is negative.

Sample size	All experiments were performed in two to five replicates. Experiments with two replicates were replicated in different cell lines . No statistical method was used to determine sample size. Sample sizes were based upon previously reported data.
Data exclusions	No data were excluded from the analysis
Replication	All replications were succesfull.
Randomization	The samples or the cells were randomized to be examined.
Blinding	Not relevant.

Reporting for specific materials, systems and methods

We require information from authors about some types of materials, experimental systems and methods used in many studies. Here, indicate whether each material, system or method listed is relevant to your study. If you are not sure if a list item applies to your research, read the appropriate section before selecting a response.

Materials & experimental systems

n/a	Involved in the study	
☐	☒	Antibodies
☐	☒	Eukaryotic cell lines
☒	☐	Palaeontology
☒	☐	Animals and other organisms
☐	☒	Human research participants
☒	☐	Clinical data

Methods

n/a	Involved in the study	
☒	☐	ChIP-seq
☒	☐	Flow cytometry
☒	☐	MRI-based neuroimaging

Antibodies

Antibodies used

Western blot was performed using antibodies against DHX36 (1:1000, Abcam Ab70269), DHX9 (1:1000, Abcam Ab54593), DDX3X (1:1000, Santa Cruz sc-365768), LARP1 (1:1000, Bethyl A302-087A), hnRNP H/F (1:1000, Abcam Ab10689), KSRP (1:500, Bethyl A302-022A), E2F1 (1:500, Santa Cruz sc-251), eIF4A (1:500, Santa Cruz sc-50354), PERK (1:1000, Cell Signaling Technology 3192), Histone H3 (1:1000, Cell Signaling Technology 4499), EEA1 (1:500, Santa Cruz sc-53939), RPS6 (1:1000, Santa Cruz sc-74459), RPL22 (1:1000, Novus Bio NBP1-06069), GAPDH (1:1000, Santa Cruz sc-32233), γH2AX (1:1000, Millipore 05-636), Flag (1:1000, Sigma F3165-2MG), USP1 (1:600, ProteinTech 14346-1-AP), Ubiquitin (1:1000, Cell signaling Technology 3936), Puromycin (1:1000, Millipore, MABE343), PARP (1 :1000, Cell signaling 9542), Caspase-3 (1 :1000, Cell signaling 8G10), Anti-Rabbit IgG (1:5000, Ozyme 7074S), Anti-Mouse IgG (1:5000, Ozyme 7076S). Immunofluoresence was performed with γ-H2AX (JBW301 Millipore 05-636; 1:500), 53BP1 (Cell Signaling 2675; 1:200) and with 1 µg per slide of BG4 antibody. RIP was performed with hnRNP H/F (10 µg, Abcam Ab10689), DHX36 (5 µg, Abcam Ab70269) or BG4 antibodies (0,5 µg). BG4 was expressed from the pSANG10-3F-BG4 plasmid (Addgene #55756).

Validation

We validated the specificity of the antibodies used by siRNA-mediated experiments and western-blot analysis. All the antibodies were used according to the manufacturer instructions and were supported by multiple publications.

Eukaryotic cell lines

Policy information about cell lines

Cell line source(s)

U251-MG ECACC #09063001; LN18 ATCC #CRL-2610; U87 SIGMA #89081402-1VL

Authentication

Commercial cancer cell lines were used without further authentication

Mycoplasma contamination

All used cell lines are free of mycoplasma contamination

Commonly misidentified lines
(See ICLAC register)

No commonly misidentified cell lines were used in the study

Human research participants

Policy information about studies involving human research participants

Population characteristics

Designation Gli22b: Gender and age: Male, 68; Diagnosis and tumour type: Glioblastoma (grade IV), Mutation: Not known, Treatment categories: not known.
Designation Gli25: Gender and age: Male, 66; Diagnosis and tumour type: Glioblastoma (grade IV), Mutation: Not known, Treatment categories: not known.
Designation Gli26: Gender and age: Female, 69; Diagnosis and tumour type: Glioblastoma (grade IV), Mutation: Not known, Treatment categories: not known.
Designation LGG 103: Gender and age: Male, 36; Diagnosis and tumour type: Oligodendroglioma Grade II , Mutation: Total loss 1p19q, IDH1 mutation R132H, Treatment categories: Resection
Designation LGG 112: Gender and age: Female, 38; Diagnosis and tumour type: Oligodendroglioma Grade II , Mutation: Total loss 1p19q, IDH1 mutation R132H, Treatment categories: Resection
Designation LGG 110: Gender and age: Female, 43; Diagnosis and tumour type: Astrocytoma Grade II, Mutation: No loss 1p19q, IDH1 mutation R132H, Treatment categories: Resection
Designation LGG 141: Gender and age: Female, 25; Diagnosis and tumour type: Astrocytoma Grade II, Mutation: No loss 1p19q, IDH1 mutation R312H, Treatment categories: Resection

Recruitment

Proteins were extracted from patients diagnosed for gliomas (high or low grade) at the Montpellier hospital. There is no obvious self-selection bias for the recruitement of these patients who are recruited at the National level and are not family related.

Ethics oversight

We declare that all methods used in this article were carried out in accordance with relevant guidelines and regulations of French Institut National de la Santé et de la Recherche Médicale (INSERM). We also declare that all experimental protocols were approved by INSERM. Glioma samples were obtained from the « Centre de Ressources Biologiques » of the Montpellier Hospital (Collection NEUROLOGIE (8) DC-2013-2027 / DC-2010-1185 /Authorization AC-2017-3055 / Research Protocol P487). We declare

that informed and written consents were obtained from all samples used in the article. Gliomas were graded by a neuropathologist (Pr V Rigau, CHU Montpellier)"

Note that full information on the approval of the study protocol must also be provided in the manuscript.
